# Psychosocial Workplace Factors and Healthcare Utilization: A Study of Two Employers

**DOI:** 10.15171/ijhpm.2017.132

**Published:** 2017-11-26

**Authors:** Jessica Allia Williams, Orfeu Buxton, Jesse Hinde, Jeremy Bray, Lisa Berkman

**Affiliations:** ^1^Harvard Center for Population & Development Studies, Cambridge, MA, USA.; ^2^The University of Kansas Medical Center, Department of Health Policy and Management, Kansas City, KS, USA.; ^3^Department of Biobehavioral Health, Pennsylvania State University, State College, PA, USA.; ^4^Division of Sleep Medicine, Harvard Medical School, Boston, MA, USA.; ^5^Department of Social and Behavioral Sciences, Harvard T.H. Chan School of Public Health, Boston, MA, USA.; ^6^Department of Public Policy, University of North Carolina at Chapel Hill, Chapel Hill, NC, USA.; ^7^RTI International, Research Triangle Park, NC, USA.; ^8^Department of Economics, University of North Carolina Greensboro, Greensboro, NC, USA.; ^9^Harvard Center for Population and Development Studies, T.H. Chan Harvard School of Public Health, Cambridge, MA, USA.

**Keywords:** Healthcare Utilization, Psychosocial Workplace Factors, Work-Family Conflict, ER Visits

## Abstract

**Background:** While a large literature links psychosocial workplace factors with health and health behaviors, there is very little work connecting psychosocial workplace factors to healthcare utilization.

**Methods:** Survey data were collected from two different employers using computer-assisted telephone interviewing as a part of the Work-Family Health Network (2008-2013): one in the information technology (IT) service industry and one that is responsible for a network of long-term care (LTC) facilities. Participants were surveyed four times at six month intervals. Responses in each wave were used to predict utilization in the following wave. Four utilization measures were outcomes: having at least one emergency room (ER)/Urgent care, having at least one other healthcare visit, number of ER/urgent care visits, and number of other healthcare visits. Population-averaged models using all four waves controlled for health and other factors associated with utilization.

**Results:** Having above median job demands was positively related to the odds of at least one healthcare visit, odds ratio [OR] 1.37 (P<.01), and the number of healthcare visits, incidence rate ratio (IRR) 1.36 (P<.05), in the LTC sample. Work-to-family conflict was positively associated with the odds of at least one ER/urgent care visit in the LTC sample, OR 1.15 (P<.05), at least one healthcare visit in the IT sample, OR 1.35 (P<.01), and with more visits in the IT sample, IRR 1.35 (P<.01). Greater schedule control was associated with reductions in the number of ER/urgent care visits, IRR 0.71 (P<.05), in the IT sample.

**Conclusion:** Controlling for other factors, some psychosocial workplace factors were associated with future healthcare utilization. Additional research is needed.

## Background

Psychosocial workplace factors are “interactions between and among work environment, job content, organizational conditions and workers’ capacities, needs, culture, personal extra-job considerations that may, through perceptions and experience, influence health, work performance and job satisfaction.”^1^ An increasingly robust literature links psychosocial workplace factors and other working conditions, such as long hours, to poor health outcomes and behaviors.^2-8^ For example, Job strain, a combination of high psychological demands at work combined with low control over how work is accomplished, has long been associated with cardiovascular disease, work-related musculoskeletal disorders, and depressive symptoms in many, though not all, studies.^9-17^ Another frequently cited workplace factor, work-family conflict, describes a situation where work and family roles are inconsistent in a way that makes those roles more difficult to fulfill and has been associated with obesity, poor sleep quality, cardiovascular disease, depressive symptoms, sleep quantity, and sleep quality.^18-23^ Schedule control, how much control an individual has over the arrangement of their work hours, has also been linked to wellbeing.^24^ Other workplace factors have direct connections to health as well, with many reviews published over time.^8,25-28^

Despite the link to health outcomes, there is comparatively less work connecting psychosocial workplace factors to healthcare utilization—especially using validated measures and more than one psychosocial factor. Aldana and colleagues concluded that having a workplace with health supportive leadership and health-related policies to improve worker health may result in lower medical costs, but few other psychosocial factors were accounted for in the analysis.^29^ One study using two employers and medical claims data did not find robust associations with work environment measures, but these measures were not validated.^30^ Another study found higher baseline co-worker support was associated with more doctor’s visits but not with other types of utilization.^31^ Others have found that personal control perceptions and workload demand predicted increased utilization over 5 years after controlling for previous utilization but not for health and social factors.^32^ Modrek and colleagues found that both inpatient and outpatient mental health visits increased for all workers after 2009 and were even higher for workers at plants with more layoffs (higher job insecurity).^33^ Other authors have also found general increases in utilization after layoffs but with differences by type of utilization.^34^

Using previous literature regarding health and healthcare utilization, this manuscript investigates several different workplace psychosocial factors simultaneously. Job insecurity, the perceived likelihood of losing your job, has been previously associated with healthcare use—potentially because of health needs or because employment and health insurance are linked in the U.S.^34^ The number of work hours has also been associated with poorer health outcomes and may affect ability to use healthcare.^24^

There are two primary ways that psychosocial workplace factors may affect healthcare utilization. Better psychosocial working conditions may improve health, in turn reducing future healthcare utilization. However, better psychosocial working conditions may indicate greater flexibility—reducing employee opportunity costs (value of next highest value alternative, here, work) of obtaining healthcare—and so be associated with greater future healthcare utilization. For example, low levels of schedule control may lead to reduced use of regular healthcare (but potentially increasing emergency care use) after controlling for health and other factors.^35,36^ Additionally, psychosocial factors may alter an individual’s discount rate (current worth of future sum of money), changing their short- and long-term demand for health and healthcare.^37^

We expect psychosocial workplace factors that increase the opportunity or time costs of utilization, such as high demands, low control, and work-to-family conflict, will be negatively associated with utilization after controlling for health status. We further expect that psychosocial workplace factors that decrease the opportunity or time costs, such as schedule control, will be positively associated with utilization after controlling for health status.

## Methods

### Participants and Setting

Data for these analyses were obtained from employers in two different industries in the United States as a part of the Work, Family and Health Network (WFHN): an information technology (IT) focused unit of a Fortune 500 company (hereafter IT setting) and a chain of long-term care facilities (hereafter LTC setting).^38^ The WFHN study was a longitudinal, group-randomized trial of a workplace intervention designed to reduce work-family conflict and improve health and family processes. Intervention components included supervisor training and interactive work re-design sessions with employees.^39^ While this study does not examine intervention effects from the WFHN study, we include controls for the study design (sites were randomized using coin-based and adaptive randomization techniques). The study period lasted from 2008-2013.

The IT setting had 56 unique work groups and 30 separate facilities (referred to as sites hereafter). All employees were included.^38,40^ Workers were mainly located in Colorado and Ohio, but some included workers were telecommuters. In the LTC setting, we included all direct care staff who were employed 24 or more hours per week and who had at least some day shifts to ensure a relatively homogenous sample of worker types across facilities.^38^ Direct care staff included nurses, nursing assistants, and supervisory staff. LTC facilities were located in the Northeast spanning all 6 New England states. Additional descriptive information on IT and LTC samples are available in peer- reviewed papers; documentation is also available online.^23,41,42^

In both settings, the study was introduced by management through mass communication and small-group meetings with supervisors. Workers were recruited directly by trained survey staff to participate in a 60-minute interview that included: (1) a survey collected using computer-assisted personal interviews, and (2) a health assessment (height, weight, and blood pressure measurements, dried blood spot). Workers received $20 to complete the survey and health assessment. Interviews were conducted at baseline, and 6, 12, and 18 months post-baseline. Employee responses were kept confidentially by the research team. The data collection for sites were staggered over time due to study resource constraints and to accommodate the business needs of the industry partners. Baseline response rates were 70% (823 of 1171 eligible workers) in the IT setting and 89% (1524 of 1783 eligible workers) in the LTC setting.^38^

### Measurement

Data were aligned so that covariates from one survey were used to predict utilization in the 6 months after that survey. For example, baseline work characteristics were used to predict utilization in the 6 months after baseline (as measured by the retrospective question on the 6-month follow-up survey). The same is true for successive waves.

### Outcomes: Healthcare Utilization

Although the measures of utilization were self-reported, they were captured using a standardized instrument (Economic Form 90) that has been used to assess costs of interventions and healthcare use among in alcohol dependence and substance use studies.^43^ Individuals were asked separately about the number of visits made to an emergency room (ER) or urgent care center (“[d]uring the past 6 months, have you made a visit to the ER or urgent care treatment facility for health treatment? How many visits did you make?”) and the number of visits made to any other healthcare provider (“[d]uring the past 6 months, have you visited any other healthcare professionals to receive outpatient treatment or counseling during the past 6 months? How many visits did you make?”). Responses for each type of utilization were kept as count variables (the number of visits/nights) and dichotomized into indicators for having at least one visit/night. All measures of utilization included utilization related to mental health and substance abuse.

### Psychosocial Workplace Factors

We included several measures of the psychosocial workplace in the analyses: schedule control, job strain, work-to-family conflict, and job security (Table 1). Schedule control, degree to which employees control the arrangement of their work hours, was the average score from a validated 8-item scale—each item was rated on a 5-point scale with higher numbers indicating greater control (very little = 1, little = 2, a moderate amount = 3, much = 4, very much = 5).^44^ Cronbach α values at baseline were .79 for the IT setting and .65 for the LTC setting.

**Table 1:** Descriptive Statistics for the Baseline LTC Setting and IT Settings

**Variable**	**LTC Sample**	**IT Sample**
	**Mean (SD) or Percent**	**Mean (SD) or Percent**
Future ER/urgent care visits (if >0 visits)	1.58 (1.25)	1.45 (1.69)
Future other healthcare visits (if >0 visits)	3.87 (4.58)	3.91 (5.71)
Schedule control (1-5 scale)	2.68 (0.74)	3.59 (0.68)
Job demands (1-5 scale)	3.83 (0.73)	3.61 (0.70)
Decision authority (1-5 scale)	3.47 (0.77)	3.83 (0.69)
Work-to-family conflict (1-5 scale)	2.76 (0.89)	3.06 (0.95)
Job insecurity (% with insecurity)	7.79%	34.60%
Tenure (years)	7.66 (7.14)	14.05 (9.02)
Work hours (this job)	37.33 (7.22)	45.29 (5.42)
Psychological distress	11.87 (4.25)	10.84 (3.34)
BMI (by category)		
Normal or underweight (BMI <25)	28.35%	31.43%
Overweight (BMI ≥25 and BMI <30)	30.17%	39.24%
Obese (BMI ≥30)	41.48%	29.32%
Age (y)	39.89 (12.15)	46.61 (8.52)
Hypertension	24.94%	27.43%
Smokes	27.86%	6.33%
Diabetes	8.27%	7.17%
Heart attack or MI	1.46%	1.48%
Stroke	1.58%	1.27%
Hours in bed	7.27 (1.43)	7.31 (0.95)
Annual personal income		
≤$9999	2.31%	--
$10 000-$19 999	11.56%	--
$20 000-$29 999	33.82%	--
$30 000-$39 999	20.68%	--
$40 000-$49 999	12.53%	--
$50 000-$59 999	9.85%	--
>$60 000	9.25%	--
Annual personal income		
<$60 000	--	3.38%
$60 000-$79 999	--	26.16%
$80 000-$99 999	--	37.55%
$100 000-$119 999	--	24.26%
>$120 000	--	8.65%
Married/living with partner	64.11%	81.86%
Number of children	1.11 (1.22)	1.03 (1.06)
Household Size	3.20 (1.52)	3.00 (1.37)
Provides care outside work	29.93%	23.84%
Female	92.46%	37.76%
Educational attainment		
Less than high school graduate	5.96%	0.00%
High school graduate	32.73%	1.90%
Some college/technical school	50.12%	19.20%
College graduate	11.19%	78.90%
Health insurance status		
Not eligible, not enrolled	3.41%	Not available
Eligible, not enrolled	25.79%	Not available
Eligible, enrolled	61.68%	Not available
No data available	9.12%	Not available
Job title hierarchy		
Administrative personnel/coordinators	Not available	5.49%
Level I/II contributors	Not available	34.18%
Lead/staff/principal contributors	Not available	60.34%

Abbreviations: ER, emergency room; LTC, long-term care; BMI, body mass index; IT, information technology; SD, standard deviation.

Job strain, a combination of high psychological job demands and low decision authority, was measured with the Job Content Questionnaire.^14^ The Job Content Questionnaire measure has been used extensively in the literature and has been associated with negative health outcomes, such as cardiovascular disease.^45^ Validated psychological job demands and decision authority scales were each measured with multiple items; the average of the items was used to create individual scale scores. Responses for each item were on a 5-point scale from strongly disagree to strongly agree. Psychological job demands (higher scores reflect greater job demands) was measured with three items: (1) “You do not have enough time to get your job done,” (2) “Your job requires very fast work,” and (3) “Your job requires very hard work.”^46^ Its baseline alpha values of 0.71 in the IT setting and 0.55 in the LTC setting. Decision authority (higher score indicate greater decision authority) was measured with three items: (1) “Your job allows you to make a lot of decisions on your own,” (2) “On your job, you have very little freedom to decide how you do your work,” and (3) “You have a lot of say about what happens on your job.” It had baseline Cronbach α values of .58 in the IT setting and .60 in the LTC setting. Because psychological job demands and decision authority are valid sub-scales of job strain and that each component may have independent associations with healthcare utilization, we include them as separate measures in the model. Because the measures are relative within the sample (High and Low Job Strain, Active, Passive jobs are defined relative to median splits), we use indicators to denote whether an individual had above median (of each setting respectively) job demands (4 for LTC, 3.66 for IT) and/or below median decision authority (3.66 for LTC, 4 for IT).^47^

Work-to-family conflict was measured using the mean of the 5-item construct, a validated and widely used scale (Work-Family Conflict Scale).^48^ Each item was measured on a 5-point scale (strongly disagree to strongly agree) with higher scores indicating higher levels of conflict: “[t]he demands of your work interfere with your family or personal time,” “[t]he amount of time your job takes up makes it difficult to fulfill your family or personal responsibilities,” “[t]hings you want to do at home do not get done because of the demands your job puts on you,” “[y]our job produces strain that makes it difficult to fulfill your family or personal duties,” “[d]ue to your work-related duties, you have to make changes to your plans for family or personal activities.”^48^ Cronbach α values at baseline were .91 for the IT setting and .88 for the LTC setting.

A measure of job security was used to assess the risk of losing the respondent’s current job. This measure (“[t]hinking about the next 12 months, how likely do you think it is that you will lose your job or be laid off?”) was adapted from the General Social Survey and was dichotomized to indicate greater likelihood of losing a job.^49^ Study data were collected during an economic downturn and could greatly influence stress and well-being. In addition to psychosocial workplace factors, we included weekly work hours to control for workload and years on the job (tenure).

### Additional Covariates

To reduce the potential bias in the empirical model, we include several covariates that may be correlated with the participants’ health status, psychosocial workplace factors and healthcare use: psychological distress, body mass index (BMI), multiple indicators of chronic conditions, smoking status, sleep, age, and gender. The Kessler-6 index was used to measure psychological distress, with a potential range of 6-30 and higher scores indicating higher levels of distress.^50^ BMI was computed from physical measures of participants’ weights and heights and was divided into categories as specified by the Centers for Disease Control and Prevention (CDC): underweight/normal weight, overweight, and obese.^51^ Additionally, separate indicators for chronic conditions were added to the model for whether participants had ever been told that they had high blood pressure, diabetes, stroke, heart attack or coronary or myocardial infarction. Participants were coded as being current smokers if they reported smoking cigarettes “some days” or “daily.” Number of hours in bed were also included a measure of participant sleep.

Additionally, we include several other covariates that may be correlated with psychosocial workplace and healthcare use: annual personal income in categories, educational attainment, being married or living with a partner (dichotomous), the number of children under age 18, and household size. A dichotomous indicator for spending at least 3 hours a week caring for an adult relative inside or outside of home during the previous 6 months was included to measure caregiving as these roles may be correlated with psychosocial workplace factors or affect the healthcare use.

To account for the study design, we include a dichotomous indicator for workers in sites randomized to the intervention group and variables used in the randomization scheme. Other authors have explored the effects of the intervention.^52-54^ We also included dichotomous indicators for survey waves.

Some variables were only available for one of the settings. In the LTC setting, eligibility and enrollment status for employer-sponsored health insurance were available from administrative data and included in the model as a categorical variables. Given the differing healthcare systems in different markets, state indicators were also included. For the IT setting, information on the relative status of job classifications and controls for a co-occurring merger were included as covariates.^55^

Because the exact nature of the association between utilization and workplace outcomes is uncertain, we used interactions of the workplace variables (job demands and job control, work-to-family conflict and schedule control) as sensitivity analyses. To address the concern that self-reported health was inadequately measured, we used the LTC setting to run a sensitivity analysis that used biomarkers combined to form a cardio-metabolic risk score, in place of the other health measures. We also ran sensitivity analyses that included an indicator for heavy alcohol use since that might have be related to utilization.^56^

### Statistical Analyses

Only individuals who answered all of the surveys were included in the main analyses. Models were also estimated including individuals who completed all survey waves but were missing data values in some waves and for those who completed at least one survey wave are shown in Supplementary file 1. In results not shown, we also ran models using a method of multiple imputation (multivariate normal with 10 imputations and bootstrapped 95% CIs); these results were quite similar as well. For the binary outcomes of receiving any care, we used population-averaged logit models. For numbers of visits we used generalized linear models (negative binomial family with log link). We adjusted standard errors for clustering by individual. Results are reported using odds ratios (ORs) for dichotomous outcomes and incidence rate ratios (IRRs) for numbers of visits. All results are shown for the workplace variables, but only results statistically significant at the 5% level are discussed. All statistical analyses were run using STATA 13.1.^57^

## Results

### Participants

In the LTC setting, 1524 individuals answered the baseline survey; 931 individuals completed all four waves of the survey. After omitting individuals with missing data, the final sample included 882 individuals. In the IT setting 823 individuals answered the baseline survey; 609 individuals completed all four waves of the survey and 490 people remained in the sample after omitting those with missing data.

### Descriptive Data

Descriptive data are discussed with reference to the baseline survey for simplicity (see Table 1), although they were answered at each survey wave. In the LTC setting, 17.64% of participants reported having at least one ER/urgent care visit during the 6 months following the baseline survey. More participants, 28.50%, reported having at least one other healthcare visit during the same timeframe. In the IT setting, for the same relative time period, 7.76% of participants reported having at least one ER/urgent care visit and 38.57% reported having at least one other healthcare visit. Distributions of both types of visits in both samples were quite skewed with long right tails—enough that the standard deviations are greater than the means for some variable.

### Multivariable Regression Results

#### Outcome: Any Visits

In the LTC setting, the results for having any ER/urgent care or other healthcare are given in Table 2, column 1. Greater work-to-family conflict was associated with higher odds of having any ER/urgent care visits (*P* = .049). For example, moving from the average value of work-to-family conflict to 1 standard deviation above average would be associated with a 1.75 percentage point increase in the probability of having at least one ER/urgent care visit. None of the other workplace psychosocial factors were statistically significant at the 5% level.

**Table 2:** Results From the Analyses of Having any Visits (LTC Sample)

	**Any Emergency/Urgent Care Visits**	**Any Other Healthcare**
	**OR (95% CI)**	**OR (95% CI)**
Schedule control	1.03 (0.86, 1.22)	0.97 (0.83, 1.13)
Job demands		
Below median	Reference	Reference
Above median	1.04 (0.82, 1.32)	1.37^b^ (1.12, 1.68)
Decision authority		
Above median	Reference	Reference
Below median	0.94 (0.73, 1.22)	0.90 (0.72, 1.12)
Work-to-family conflict	1.15^a^ (1.00, 1.32)	1.05 (0.93, 1.19)
Job insecurity		
No	Reference	Reference
Yes	0.92 (0.57, 1.48)	0.74 (0.52, 1.06)
Tenure (y)	0.98 (0.96, 1.00)	0.98 (0.96, 1.01)
Work hours (this job)	0.99 (0.97, 1.01)	0.99 (0.97, 1.01)
No. of observations	2466	2463
No. of individuals^c^	822	821

Abbreviations: LTC, long-term care; OR, odds ratio. Notes: Regression also controlled for psychological distress, body mass index category, age, hypertension, smokes, diabetes, heart disease, stroke, cancer, hours in bed, annual personal income in categories, married/living with partner, number of children, household size, provides care outside work, male, educational attainment in categories, employer-sponsored health insurance status, state, intervention group, time and a constant. ^a^ *P* < .05; ^b^*P* < .01. ^c^ Sample size differs because of missing utilization information.

The odds of having any other healthcare visit were increased by a factor of 1.37 for those with above median job demands in the LTC setting (Table 2, column 2, *P* = .002). In other words, having above median job demands was associated with a 5.8% percentage point increase in the probability of having at least one other healthcare visit in the next 6 months.

In the IT setting, the results for having any ER/urgent care or other healthcare are given in Table 3, column 1. Having greater schedule control was associated with lower odds of having at least one ER/urgent care visits and was only marginally statistically significant (*P* = .051).

**Table 3:** Results From the Analyses of Having any Visits (IT Sample)

	**Any Emergency/Urgent Care Visits**	**Any Other Healthcare**
	**OR (SE)**	**OR (SE)**
Schedule control	0.73 (0.53, 1.00)	0.96 (0.79, 1.18)
Job demands		
Below median	Reference	Reference
Above median	0.84 (0.53, 1.33)	1.01 (0.78, 1.32)
Decision authority		
Above median	Reference	Reference
Below median	0.96 (0.61, 1.51)	0.77 (0.59, 1.01)
Work-to-family conflict	0.95 (0.70, 1.30)	1.35^a^ (1.14, 1.61)
Job insecurity		
No	Reference	Reference
Yes	0.88 (0.54, 1.44)	0.97 (0.75, 1.26)
Tenure (y)	0.99 (0.95, 1.02)	0.99 (0.98, 1.01)
Work hours (this job)	1.01 (0.97, 1.06)	0.98 (0.96, 1.01)
No. of observations	1470	1470
No. of individuals	490	490

Abbreviations: IT, information technology; OR, odds ratio; SE, standard error. Notes: Regression also controlled for psychological distress, body mass index category, age, hypertension, smokes, diabetes, heart disease, stroke, hours in bed, annual personal income in categories, married/living with partner, number of children, household size, provides care outside work, male, educational attainment in categories, job category, intervention group, time, randomization blocking factors, merger indicator, and a constant. ^a^ *P* < .05; ^b^ *P* < .01.

In the model of having any other healthcare visits for the IT setting, Table 3, column 2, work-to-family conflict was associated with higher odds of having at least one visit (OR: 1.35, *P* = .001). Moving from the mean to one standard deviation above the mean work-to-family conflict was associated with a 6.6 percentage point increase in the probability of having at least one other healthcare visit in the next 6 months.

#### Outcome: Number of Visits

Results for the number of visits outcomes are given in Table 4, column 1. In the LTC setting, none of the psychosocial workplace variables were statistically significant at the 5% level with respect to the number of ER/urgent care visits. The rates of other healthcare visits (Table 4, column 2) for participants with above median job demands were 1.36 times greater than for those with below median job demands (*P* = .01), which translates to an additional 0.34 visits over 6 months.

**Table 4:** Results From the Number of Visits (LTC Sample)

	**Any Emergency/Urgent Care Visits**	**Any Other Healthcare**
	**IRRs (SE)**	**IRRs (SE)**
Schedule control	0.95 (0.80, 1.12)	0.92 (0.78, 1.09)
Job demands		
Below median	Reference	Reference
Above median	1.15 (0.93, 1.43)	1.36^a^ (1.07, 1.73)
Decision authority		
Above median	Reference	Reference
Below median	0.92 (0.73, 1.17)	0.90 (0.69, 1.17)
Work-to-family conflict	1.06 (0.93, 1.21)	1.11 (0.97, 1.28)
Job insecurity		
No	Reference	Reference
Yes	0.96 (0.60, 1.54)	0.74 (0.50, 1.10)
Tenure (y)	0.98 (0.97, 1.00)	0.98 (0.96, 1.00)
Work hours (this job)	0.99 (0.97, 1.00)	0.99 (0.97, 1.01)
No. of observations	2466	2463
No. of individuals	822	821

Abbreviations: LTC, long-term care; SE, standard error; IRRs, incidence rate ratios. Notes: Regression also controlled for psychological distress, body mass index category, age, hypertension, smokes, diabetes, heart disease, stroke, cancer, hours in bed, annual personal income in categories, married/living with partner, number of children, household size, provides care outside work, male, educational attainment in categories, employer-sponsored health insurance status, state, intervention group, time and a constant. ^a^ *P* < .05; ^b^ *P* < .01. ^c^ Sample size differs because of missing utilization information.

In the IT setting, greater schedule control was associated with lower rates of visits to ER/urgent care centers (IRR 0.71, *P* = .03) but none of the other workplace factors were statistically significant at the 5% level (Table 5, column 1). In terms of scale, moving one standard deviation above the mean of schedule control was associated with a 0.02 decrease in visits over the next 6 months. In the model of other healthcare, rates of visits were greater for those experiencing more work-to-family conflict (Table 5, column 2, IRR 1.35, *P* = .001). This would translate to an additional 0.49 visits over the next 6 months.

**Table 5:** Results From the Number of Visits (IT Sample)

	Any Emergency/Urgent Care Visits	Any Other Healthcare
	IRRs (SE)	IRRs (SE)
Schedule control	0.71^a^ (0.52, 0.97)	1.21 (0.96, 1.53)
Job demands		
Below median	Reference	Reference
Above median	0.72 (0.44, 1.15)	0.83 (0.62, 1.11)
Decision authority		
Above median	Reference	Reference
Below median	0.94 (0.63, 1.41)	1.07 (0.83, 1.38)
Work-to-family conflict	1.01 (0.74, 1.39)	1.35^a^ (1.13, 1.61)
Job insecurity		
No	Reference	Reference
Yes	0.83 (0.54, 1.30)	0.80 (0.62, 1.03)
Tenure (y)	0.97 (0.94, 1.01)	0.98^a^ (0.96, 1.00)
Work hours (this job)	1.03 (0.99, 1.06)	0.98 (0.96, 1.01)
No. of observations	1470	1470
No. of individuals	490	490

Abbreviations: IT, information technology; SE, standard error; IRRs, incidence rate ratios. Notes: Regression also controlled for psychological distress, body mass index category, age, hypertension, smokes, diabetes, heart disease, stroke, cancer, hours in bed, annual personal income in categories, married/living with partner, number of children, household size, provides care outside work, male, educational attainment in categories, employer-sponsored health insurance status, state, intervention group, time and a constant. ^a^ P < .05; ^b^ P < .01.

#### Results of Sensitivity Analyses

Using interactions of the workplace variables and controlling for heavy alcohol use did not significantly alter the results. Additionally, using measured biomarkers in the form of a cardio-metabolic risk score^58^ in the LTC sample, yielding essentially the same results even with a smaller sample than the original analyses.

## Discussion

### Key Results and Interpretation

Certain poor psychosocial workplace factors are positively associated with future healthcare utilization even after controlling for health and other factors in two very different employee populations. The directions of the relationship found in these analyses are the opposite of what was hypothesized. These findings suggest that stressful psychosocial working conditions may lead to increased healthcare utilization above and beyond their effect on utilization through health.

The findings also differed by sample, with some mattering more for one population of employees more than the others. Above median job demands were associated with more healthcare visits in the LTC sample but not in the IT sample. Schedule control was negatively associated with ER/urgent care utilization in only the IT sample. Given that the employee population are very different this result is not surprising. The companies represented very different sets of occupations and employees, different work factors may have been binding for the different groups. The LTC company generally had more positions that require physical effort, less education, and more interaction with patients. The IT company had more positions that require less interaction with outside individuals, less physicality, and higher education requirements. In addition to different people selecting into these two types of work, the daily stressors for the positions are likely to be quite different, as we saw with the descriptive statistics. Schedule control was likely especially important for IT workers compared to LTC workers because they worked more hours. Other work has found differences in the level and relative risk faced by employees in different occupations.^59,60^

The schedule control finding fits into the literature of healthcare demand because all else equal, individuals with greater schedule control should be more likely to access care more regularly and not have to go to ER/urgent care centers. While we could not compare our results to previous studies that did not use standard psychosocial measures, the finding from the LTC sample that demands positively related to utilization matched the direction of effect of the results by Gartner and colleagues.^32^ Our lack of finding an association with job insecurity does not match pervious work. Given that our analyses controlled for a wide array of personal and social factors, including previous healthcare utilization, it is not surprising that the size of the associations between workplace factors and utilization was smaller than found in previous work.

Is more healthcare a good thing or a bad thing? Higher utilization could mean that people are sicker (a bad outcome) or that they do not have barriers to accessing care (a good outcome). Without more information about the health status of workers and detailed healthcare information about why care was accessed, we cannot shed light on the “other utilization” variable used as an outcome. On the other hand, ED/ER visits are almost always “bad” in that they indicate an acute urgent condition, which is never good, or a non-urgent condition that was not adequately dealt with using a lower level of care indicating inappropriate access. Depending on the type of utilization, future work could investigate whether changing the psychosocial factors, such as schedule control, might change utilization with the goal of making healthcare more efficient and employees healthier.

## Limitations

The primary limitations of theses analyses are that they reflect only two employers and that utilization data are self-reported. Although self-reported healthcare utilization data have variable accuracy, the recall period of 6 months and the relatively good health status of the sample increase the likelihood of having accurate estimates, especially for ER/urgent care visits.^61-63^ Administrative claims data were not available. We also did not have information about health conditions, such as asthma and arthritis, which may have resulted in increased utilization but were not included in the surveys. Including these conditions might make it more likely that the marginal association of workplace factors would be closer to the null. Although both employers offered health insurance to their employees, we were unable to control for aspects of plan design for anyone and insurance status for those who did not have employer sponsored coverage in the LTC sample—in the IT setting we were unable to control for insurance status because we did not have access to that information. Additionally, individuals may seek out a health professional for preventive care or to address a health problem—our analyses cannot distinguish between these types of healthcare visits. The Cronbach α values for job strain measures were also on the low side. However, these measures allow comparisons to other work and have been shown to be valid in other samples.

## Conclusion

While there is little work on the role of psychosocial workplace factors and healthcare utilization, existing studies have not shown the relationships shown in this study.^30^ Our results, obtained using panel data from two sets of employees and validated measures of the psychosocial environment, that having above median job demands and higher work-to-family conflict lead to greater healthcare utilization suggest that improving these psychosocial workplace factors may pay off for employers through more than just improved health—they may lead to changes in utilization as well. Additional work should be done to evaluate the marginal role of psychosocial factors in healthcare utilization and studies of healthcare demand should consider incorporating measures of the psychosocial work environment to fully understand the role it plays.

## Ethical issues

Approval was obtained from all participating WFHN sites. These include the University of Minnesota (Minneapolis, MN, USA), Penn State University (State College, PA, USA), Harvard University (Cambridge, MA, USA), Portland State University (Portland, OR, USA), Purdue University (Cambridge, MA, USA), Kaiser Permanente’s Center for Health Research (Portland, OR, USA), RTI International (Research Triangle Park, NC, USA), and University of Southern California (Los Angles, CA, USA).

## Competing interests

No potential conflicts of interest exist for all authors in the conception, implementation, and analysis of this research.

## Authors’ contributions

Conception and design: JW, LB; Acquisition of data: All; Analysis and interpretation of data: All; Drafting of the manuscript: JW; Critical revision of the manuscript for important intellectual content: All; Statistical analysis: JW; Obtaining funding: LB, OB, JB; Administrative, technical, or material support: JH; Supervision: LB.

## Authors’ affiliations

^1^Harvard Center for Population & Development Studies, Cambridge, MA, USA. ^2^The University of Kansas Medical Center, Department of Health Policy and Management, Kansas City, KS, USA. ^3^Department of Biobehavioral Health, Pennsylvania State University, State College, PA, USA. ^4^Division of Sleep Medicine, Harvard Medical School, Boston, MA, USA. ^5^Department of Social and Behavioral Sciences, Harvard T.H. Chan School of Public Health, Boston, MA, USA. ^6^Department of Public Policy, University of North Carolina at Chapel Hill, Chapel Hill, NC, USA. ^7^RTI International, Research Triangle Park, NC, USA. ^8^Department of Economics, University of North Carolina Greensboro, Greensboro, NC, USA. ^9^Harvard Center for Population and Development Studies, T.H. Chan Harvard School of Public Health, Cambridge, MA, USA.

## Funding

Funding for the WFHN research was provided by Eunice Kennedy Shriver National Institute of Child Health and Human Development, Rockville, MD, USA (grants # U01HD051217, U01HD051218, U01HD051256, U01HD051276); National Institute on Aging, Baltimore, MD, USA (grant # U01AG027669); Office of Behavioral and Social Sciences Research National Institute for Occupational Safety and Health, Bethesda, MD, USA (grants # U01OH008788, U01HD059773); Additional funding from National Heart, Lung, and Blood Institute, Bethesda, MD, USA (grant #R01HL107240); William T. Grant Foundation, New York City, NY, USA; Alfred P. Sloan Foundation, New York City, NY, USA; and the US Department of Health and Human Services Administration for Children and Families, Washington, DC, USA. JAW was supported by the Robert Wood Johnson Foundation, Princeton, NJ, USA Health & Society Scholars program. Some results were presented at the Robert Wood Johnson Health & Society Scholars 2014 meeting in Detroit, MI, USA.

## Supplementary files


Supplementary file 1 contains Tables S1-S6.

## Key messages

**Implications for policy makers**

- We know that psychosocial workplace factors affect workers’ health and wellbeing. This manuscript also shows that in addition to health effects, certain psychosocial workplace factors may affect individuals’ use of healthcare services.
- Job risks differ by industry and the relative importance of various psychosocial workplace factors may also differently affect healthcare use by industry and employment type.

**Implications for the public**

Psychosocial workplace factors, such as having high job demands and work-to-family conflict, may negatively affect your health. This manuscript also shows that they may change your use of healthcare services after accounting for the health effects. The links between the factors and use of healthcare services differed based on job characteristics and by the type of work—information technology (IT) or long-term care (LTC). While not fully conclusive, the results suggest that more research should be done to determine whether employers and workers can take steps to mitigate on-the-job risks from poor psychosocial working environments.
