# Supplementary material for: Psychosocial Workplace Factors and Healthcare Utilization: A Study of Two Employers
Source: Int J Health Policy Manag. 2017 Nov 26;7(7):614–22. doi: 10.15171/ijhpm.2017.132 (PMC6037501; doi:10.15171/ijhpm.2017.132)
Supplement: Supplementary file 1 — contains Tables S1-S6. [file ijhpm-7-614-s001.pdf]

Table S1. Descriptive Statistics for all respondents in the LTC setting

	LTC<br>Wave 1<br>(n=1524)[1]	LTC<br>Wave 2<br>(n=1275)[1]	LTC<br>Wave 3<br>(n=1083)[1]
**Variable**	*Mean (SD) or Percent*	*Mean (SD) or Percent*	*Mean (SD) or Percent*
Percent with at least one Future ER/Urgent Care Visit[2]	18.5%	17.8%	17.0%
Number of Future ER/Urgent Care Visits (if > 0 visits)[2]	1.6 (1.9)	1.5 (1.6)	1.7 (1.9)
Percent with at least one Future Other Healthcare[2]	28.2%	25.1%	22.6%
Future Other Healthcare Visits (if > 0 visits)[2]	4.1 (4.8)	4.4 (5.8)	4.6 (5.5)
Schedule Control (1-5 scale)	2.65 (0.7)	2.6 (0.8)	2.6 (0.8)
Job Demands (1-5 scale)	3.8 (0.8)	3.8 (0.7)	3.75 (0.7)
Decision Authority (1-5 scale)	3.5 (0.8)	3.4 (0.7)	3.5 (0.7)
Work-to-Family Conflict (1-5 scale)	2.8 (0.9)	2.8 (0.9)	2.7 (0.9)
Job insecurity (1-5 scale)[3]	3.4 (0.7)	3.3 (0.7)	3.3 (0.7)
Tenure (years)	6.3 (6.5)	7.3 (6.7)	8.2 (7.0)
Work Hours (this job)	36.9 (7.2)	36.8 (7.7)	36.5 (7.4)
Psychological Distress	11.9 (4.3)	11.5 (4.2)	11.3 (4.2)
Body Mass Index (kg/m^2)	29.5 (7.0)	29.4 (6.7)	29.7 (6.8)
Age (years)	38.5 (12.5)	39.5 (12.4)	40.1 (12.4)
High Blood Pressure	24.5%	25.1%	27.1%
Smokes	30.3%	27.5%	26.0%
Diabetes	8.2%	8.2%	9.4%
Heart Attack or MI	1.5%	1.7%	2.0%
Stroke	1.3%	1.2%	1.7%
Hours in Bed	7.4 (2.1)	7.3 (1.5)	7.3 (1.5)
Annual Personal Income			
≤$9,999	2.8%	1.6%	1.8%
$10,000-$19,999	14.0%	13.3%	11.6%
$20,000-$29,999	33.7%	32.5%	33.5%
$30,000-$39,999	19.0%	19.4%	19.5%
$40,000-$49,999	12.1%	11.6%	10.5%
$50,000-$59,999	9.8%	10.1%	11.5%
>$60,000	8.5%	8.4%	8.5%
Married/living with partner	62.9%	61.3%	62.6%
Number of children	1.0 (1.2)	1.1 (1.2)	1.2 (1.2)
Household Size	3.1 (1.5)	3.1 (1.4)	3.1 (1.7)
Provides care outside work	30.1%	27.1%	24.1%
Female	91.8%	92.55%	92.24%
Educational Attainment			
Less than High School Graduate	5.78%	5.65%	6.00%
High School Graduate	32.50%	31.61%	31.67%

Some College/Technical School	49.64%	50.35%	50.42%
College Graduate	12.08%	12.39%	11.91%

1. Sample size reflects the maximum number of responses per question (some questions had fewer responses).
2. These variables are for the survey following the ones in which the characteristics were measured. For example, the baseline survey had 1522 respondents. The 6 month survey had 1275 respondents. The question about health care utilization in the 6 month survey covered the period of time just after the baseline, so there are fewer people in the estimation sample than represented here.
3. Very Likely=1, Fairly Likely=2, Not too likely=3, Not at all likely=4, Will voluntarily leave company in next 12 months= 5

Table S2. Descriptive Statistics for all respondents in the IT setting

	**IT Wave 1 (n=823)**[1]	**IT Wave 2 (n=717)**[1]	**IT Wave 3 (n=701)**[1]
**Variable**	*Mean (SD) or Percent*	*Mean (SD) or Percent*	*Mean (SD) or Percent*
Percent with at least one Future ER/Urgent Care Visit[2]	8.1%	8.4%	7.7%
Number of Future ER/Urgent Care Visits (if > 0 visits)[2]	1.4 (1.4)	1.2 (0.5)	1.2 (0.4)
Percent with at least one Future Other Healthcare[2]	37.7%	39.5%	35.3%
Future Other Healthcare Visits (if > 0 visits)[2]	3.9 (5.5)	3.6 (5.5)	4.5 (6.1)
Schedule Control (1-5 scale)	3.6 (0.7)	3.7 (0.7)	3.8 (0.7)
Job Demands (1-5 scale)	3.6 (0.7)	3.5 (0.7)	3.5 (0.7)
Decision Authority (1-5 scale)	3.8 (0.7)	3.9 (0.7)	3.9 (0.7)
Work-to-Family Conflict (1-5 scale)	3.1 (0.9)	2.9 (0.9)	2.9 (0.9)
Job insecurity (1-5 scale) [3]	2.7 (0.8)	2.6 (0.8)	2.7 (0.7)
Tenure (years)	13.7 (9.2)	14.4 (9.1)	14.88 (9.1)
Work Hours (this job)	45.4 (5.7)	45 (5.5)	45.2 (6.1)
Psychological Distress	10.9 (3.2)	10.4 (3.1)	10.3 (3.3)
Body Mass Index (kg/m^2)	28.2 (5.7)	28.1 (5.5)	28.3 (5.7)
Age (years)	45.7 (9.0)	46.6 (8.8)	47.3 (8.7)
High Blood Pressure	27.5%	27.0%	30.3%
Smokes	6.7%	5.7%	5.9%
Diabetes	7.4%	8.7%	10.0%
Previous Heart Attack or MI	1.3%	1.5%	1.6%
Stroke	1.2%	1.3%	1.7%
Hours in Bed	7.3 (1.0)	7.4 (0.9)	7.4 (1.0)
Annual Personal Income			
<$60,000	3.7%	2.9%	2.9%
$60,000-$79,999	27.5%	25.3%	21.7%
$80,000-$99,999	36.5%	36.6%	37.4%
$100,000-$119,999	24.9%	27.1%	26.8%
>$120,000	7.4%	8.1%	8.7%
Married/living with partner	79.3%	80.6%	81.2%
Number of children	1.0 (1.1)	1.0 (1.1)	1.9 (0.4)

Household Size	2.9 (1.4)	2.9 (1.3)	2.9 (1.4)
Provides care outside work	23.5%	22.7%	22.4%
Female	39.1%	37.9%	38.7%
Educational Attainment			
Less than High School Graduate	0.0%	0.0%	0.0%
High School Graduate	3.8%	2.8%	3.1%
Some College/Technical School	18.6%	18.6%	18.1%
College Graduate	77.6%	78.7%	78.7%

1. Sample size reflects the maximum number of responses per question (some questions had fewer responses).
2. These variables are for the survey following the ones in which the characteristics were measured. For example, the baseline survey had 823 respondents. The 6 month survey had 717 respondents. The question about health care utilization in the 6 month survey covered the period of time just after the baseline, so there are fewer people in the estimation sample than represented here.
3. Very Likely=1, Fairly Likely=2, Not too likely=3, Not at all likely=4, Will voluntarily leave company in next 12 months= 5

Table S3. Sensitivity Analyses of Having Any Visits (Leef Sample)

	Any Emergency/ Urgent Care Visits			Any other Healthcare		
	(1) Sample: Original, Completed all waves, completed case analysis	(1a) Sample: Completed all waves, may have missing values	(1b) Sample: Completed at least one wave, may have missing values	(2) Sample: Original, Completed all waves, completed case analysis	(2a) Sample: Completed all waves, may have missing values	(2b) Sample: Completed at least one wave, may have missing values
	Odds Ratio (SE)	Odds Ratio (SE)	Odds Ratio (SE)	Odds Ratio (SE)	Odds Ratio (SE)	Odds Ratio (SE)
Schedule Control	1.03<br>(0.09)	1.01<br>(0.09)	1.03<br>(0.08)	0.97<br>(0.07)	0.95<br>(0.07)	1.00<br>(0.07)
Above median job demands	1.04<br>(0.13)	1.00<br>(0.12)	1.07<br>(0.11)	1.37**<br>(0.14)	1.32**<br>(0.13)	1.32**<br>(0.12)
Below median decision authority	0.94<br>(0.12)	0.92<br>(0.11)	0.94<br>(0.10)	0.90<br>(0.10)	0.88<br>(0.09)	0.85+<br>(0.08)
Work-to-Family Conflict	1.15*<br>(0.08)	1.15*<br>(0.08)	1.10<br>(0.07)	1.05<br>(0.06)	1.05<br>(0.06)	1.05<br>(0.06)
Job insecurity	0.92<br>(0.22	0.85<br>(0.19)	0.94<br>(0.18)	0.74<br>(0.14)	0.80<br>(0.14)	0.79<br>(0.13)

Tenure constructed	0.98+	0.98+	0.98*	0.98+	0.98+	0.99
	(0.01)	(0.01)	(0.01)	(0.01)	(0.01)	(0.01)
Work Hours (this job)	0.99	0.99	0.99	0.99	0.99	0.99
	(0.01)	(0.01)	(0.01)	(0.01)	(0.01)	(0.01)
Psychological Distress	1.05**	1.05**	1.04**	1.03*	1.02+	1.03*
	(0.01)	(0.01)	(0.01)	(0.02)	(0.01)	(0.01)
BMI (by category)						
Normal or Underweight (BMI<25)	reference	reference	reference	reference	reference	reference
Overweight (BMI≥25 & BMI<30)	1.02	1.05	1.03	1.06	1.06	1.02
	(0.17)	(0.17)	(0.15)	(0.15)	(0.15)	(0.13)
Obese (BMI≥30)	1.40*	1.39*	1.38*	1.44**	1.35*	1.25+
	(0.22)	(0.21)	(0.18)	(0.20)	(0.18)	(0.15)
Age	1	1.00	0.99	1.01	1.01	1.00
	(0.01)	(0.01)	(0.01)	(0.01)	(0.01)	(0.00)
Hypertension	1.28	1.24	1.25+	0.95	1.02	1.01
	(0.19)	(0.18)	(0.16)	(0.13)	(0.13)	(0.12)
Smokes	1.34*	1.40*	1.39**	0.90	0.92	0.97
	(0.19)	(0.19)	(0.16)	(0.12)	(0.12)	(0.11)
Diabetes	0.61+	0.70	0.68+	1.48+	1.33	1.32
	(0.16)	(0.17)	(0.15)	(0.31)	(0.27)	(0.24)
Heart Disease	0.99	0.91	0.93	0.85	0.90	0.98
	(0.66)	(0.54)	(0.47)	(0.43)	(0.42)	(0.39)
Stroke	2.15	1.78	1.68	0.70	0.94	0.98
	(1.12)	(0.89)	(0.85)	(0.35)	(0.43)	(0.44)
Hours in Bed	0.99	0.99	0.98	0.94	0.94	0.96
	(0.04)	(0.04)	(0.04)	(0.04)	(0.04)	(0.03)
Annual Personal Income						
≤$9,999	2.48+	2.22+	1.46	0.70	1.04	0.91
	(1.31)	(1.00)	(0.58)	(0.31)	(0.40)	(0.32)
$10,000($19,999	1.31	1.10	1.08	0.86	0.85	0.95
	(0.46)	(0.36)	(0.32)	(0.23)	(0.22)	(0.23)
$20,000($29,999	1.67+	1.56	1.59+	0.75	0.79	0.90
	(0.51)	(0.45)	(0.41)	(0.17)	(0.18)	(0.19)
$30,000($39,999	1.74+	1.71+	1.63+	1.05	1.02	1.11
	(0.54)	(0.50)	(0.42)	(0.24)	(0.22)	(0.23)
$40,000($49,999	2.00*	1.73+	1.58+	1.01	0.92	1.08
	(0.64)	(0.53)	(0.43)	(0.24)	(0.21)	(0.23)
$50,000($59,999	1.83*	1.81*	1.56+	1.01	0.99	0.97
	(0.56)	(0.53)	(0.41)	(0.21)	(0.20)	(0.19)
>$60,000	reference	reference	reference	reference	reference	reference
Married/living with partner	1.01	1.00	0.99	0.99	0.93	0.89
	(0.14)	(0.13)	(0.11)	(0.12)	(0.11)	(0.09)

Number of children	1.04	0.99	0.99	0.89+	0.92	0.90+
	(0.08)	(0.07)	(0.06)	(0.06)	(0.05)	(0.05)
Household Size	0.94	0.98	0.96	1.01	1.01	1.02
	(0.07)	(0.06)	(0.05)	(0.05)	(0.04)	(0.04)
Provides care outside work	1.22	1.20	1.17	1.03	1.02	1.06
	(0.16)	(0.15)	(0.13)	(0.12)	(0.11)	(0.10)
Male	0.91	0.96	0.92	0.75	0.76	0.76
	(0.24)	(0.23)	(0.20)	(0.18)	(0.18)	(0.15)
Educational Attainment						
Less than High School Graduate	0.79	0.89	0.79	0.41**	0.43**	0.39**
	(0.32)	(0.33)	(0.25)	(0.14)	(0.13)	(0.11)
High School Graduate	1.2	1.22	1.04	0.58**	0.52**	0.47**
	(0.3)	(0.29)	(0.22)	(0.12)	(0.10)	(0.08)
Some College/Technical School	1.31	1.21	1.05	0.83	0.81	0.75+
	(0.3)	(0.26)	(0.20)	(0.15)	(0.14)	(0.11)
College Graduate	reference	reference	reference	reference	reference	reference
Constant	0.06**	0.07**	0.13**	0.56	0.57	0.59
	(0.05)	(0.06)	(0.09)	(0.44)	(0.43)	(0.37)
# Observations	2466	2659	3112	2463	2659	3112
# Individuals	822	927	1275	821	927	1275

+ p<0.10, * p<0.05, ** p<0.01

Notes: Regression also controlled for employer health insurance status, state, intervention group, and time

Table S4. Sensitivity Analyses of Having Any Visits (Tomo Sample)

	Any Emergency/ Urgent Care Visits			Any other Healthcare		
	(1) Sample: Original, Completed all waves, completed case analysis	(1a) Sample: Completed all waves, may have missing values	(1b) Sample: Completed at least one wave, may have missing values	(2) Sample: Original, Completed all waves, completed case analysis	(2a) Sample: Completed all waves, may have missing values	(2b) Sample: Completed at least one wave, may have missing values
	Odds Ratio (SE)	Odds Ratio (SE)	Odds Ratio (SE)	Odds Ratio (SE)	Odds Ratio (SE)	Odds Ratio (SE)
Schedule Control	0.73+	0.84	0.90	0.96	0.99	0.93
	(0.12)	(0.13)	(0.13)	(0.10)	(0.10)	(0.09)
Above median job demands	0.84	0.91	0.90	1.01	1.01	1.04
	(0.20)	(0.20)	(0.19)	(0.14)	(0.13)	(0.13)
Below median decision authority	0.96	0.91	0.84	0.77+	0.84	0.85
	(0.22)	(0.19)	(0.17)	(0.11)	(0.11)	(0.10)
Work-to-Family Conflict	0.95	0.93	1.00	1.35**	1.32**	1.26**

	(0.15)	(0.14)	(0.14)	(0.12)	(0.11)	(0.10)
Job insecurity	0.88	0.87	0.87	0.97	0.91	0.91
	(0.22)	(0.20)	(0.19)	(0.13)	(0.11)	(0.11)
Tenure constructed	0.99	0.98	0.98	0.99	0.99	0.99
	(0.02)	(0.02)	(0.02)	(0.01)	(0.01)	(0.01)
Work Hours (this job)	1.01	1.02	1.00	0.98	0.99	0.99
	(0.02)	(0.02)	(0.02)	(0.01)	(0.01)	(0.01)
Psychological Distress	1.02	1.03	1.04	1.02	1.03	1.02
	(0.04)	(0.03)	(0.03)	(0.02)	(0.02)	(0.02)
BMI (by category)						
Normal or Underweight (BMI<25)	reference	reference	reference	reference	reference	reference
Overweight (BMI≥25 & BMI<30)	1.22	1.05	1.14	1.10	1.07	1.20
	(0.34)	(0.27)	(0.28)	(0.17)	(0.16)	(0.17)
Obese (BMI≥30)	1.30	1.30	1.37	1.22	1.13	1.34+
	(0.39)	(0.37)	(0.37)	(0.22)	(0.19)	(0.21)
Age	1.01	1.01	1.01	1.02+	1.02*	1.02*
	(0.02)	(0.02)	(0.02)	(0.01)	(0.01)	(0.01)
Hypertension	1.36	1.11	1.13	1.11	1.21	1.19
	(0.36)	(0.28)	(0.26)	(0.17)	(0.17)	(0.16)
Smokes	2.44*	2.15*	2.23*	0.78	0.81	1.00
	(0.91)	(0.76)	(0.71)	(0.23)	(0.22)	(0.24)
Diabetes	1.57	1.42	1.53	2.19**	2.09**	1.70*
	(0.59)	(0.50)	(0.46)	(0.54)	(0.49)	(0.37)
Heart Disease	0.44	0.37	0.34	1.02	1.16	1.57
	(0.47)	(0.38)	(0.34)	(0.51)	(0.56)	(0.71)
Stroke	2.29	2.04	1.82	1.53	1.18	1.40
	(2.35)	(2.15)	(1.88)	(0.81)	(0.64)	(0.75)
Hours in Bed	1.06	1.02	1.02	1.13+	1.12+	1.10
	(0.13)	(0.11)	(0.10)	(0.08)	(0.08)	(0.07)
Annual Personal Income						
≤$9,999	0.72	0.68	0.67	0.97	0.84	0.81
	(0.92)	(0.67)	(0.58)	(0.68)	(0.46)	(0.39)
$10,000($19,999	1.10	1.17	1.22	0.99	0.81	0.79
	(1.14)	(0.81)	(0.83)	(0.54)	(0.28)	(0.26)
$20,000($29,999	0.95	1.08	1.09	1.26	0.96	0.94
	(0.90)	(0.70)	(0.70)	(0.67)	(0.30)	(0.29)
$30,000($39,999	0.44	0.67	0.80	1.22	0.94	0.96
	(0.42)	(0.44)	(0.52)	(0.66)	(0.31)	(0.30)
$40,000($49,999	1.16	1.44	1.50	1.08	0.82	0.85
	(1.18)	(1.02)	(1.04)	(0.62)	(0.31)	(0.32)
$50,000($59,999	0.51	1.36	0.98	0.58	0.55	0.59
	(0.69)	(1.36)	(0.98)	(0.43)	(0.31)	(0.30)

>$60,000	reference	reference	reference	reference	reference	reference
Married/living with partner	0.52+	0.52*	0.47**	0.68+	0.64*	0.69*
	(0.17)	(0.16)	(0.13)	(0.14)	(0.12)	(0.12)
Number of children	1.03	0.93	1.02	0.82	0.80+	0.81+
	(0.18)	(0.14)	(0.15)	(0.10)	(0.09)	(0.09)
Household Size	1.23	1.28*	1.27*	1.12	1.17	1.16
	(0.18)	(0.15)	(0.15)	(0.12)	(0.12)	(0.11)
Provides care outside work	0.75	0.85	0.88	1.32+	1.17	1.19
	(0.20)	(0.20)	(0.19)	(0.19)	(0.16)	(0.15)
Male	0.69	0.73	0.74	0.82	0.78+	0.76*
	(0.19)	(0.19)	(0.18)	(0.13)	(0.11)	(0.10)
Educational Attainment						
Grade 12 or GED	1.35	0.80	0.71	0.50	0.48	0.59
	(0.83)	(0.49)	(0.37)	(0.29)	(0.25)	(0.24)
Some College/Technical School	0.78	0.86	0.78	1.23	1.21	1.12
	(0.28)	(0.29)	(0.25)	(0.24)	(0.22)	(0.19)
College Graduate	reference	reference	reference	reference	reference	reference
# Individuals	490	582	695	490	582	695
# Observations	1470	1640	1806	1470	1640	1806

+ p<0.10, * p<0.05, ** p<0.01

Notes: Job category, Intervention group, time, randomization, and a merger indicator were also included in the model.

Table S5. Sensitivity Analyses of Number of Visits (Leef Sample)

	# Emergency/ Urgent Care Visits			# other Healthcare Visits		
	(1) Sample: Original, Completed all waves, completed case analysis	(1a) Sample: Completed all waves, may have missing values	(1b) Sample: Completed at least one wave, may have missing values	(2) Sample: Original, Completed all waves, completed case analysis	(2a) Sample: Completed all waves, may have missing values	(2b) Sample: Completed at least one wave, may have missing values
	Incidence Rate Ratios (SE)	Incidence Rate Ratios (SE)	Incidence Rate Ratios (SE)	Incidence Rate Ratios (SE)	Incidence Rate Ratios (SE)	Incidence Rate Ratios (SE)
Schedule Control	0.95<br>(0.08)	0.94<br>(0.08)	1.01<br>(0.08)	0.92<br>(0.08)	0.91<br>(0.08)	0.95<br>(0.07)
Above median job demands	1.15<br>(0.13)	1.11<br>(0.12)	1.14<br>(0.11)	1.36*<br>(0.17)	1.32*<br>(0.15)	1.37**<br>(0.15)
Below median decision authority	0.92<br>(0.11)	0.89<br>(0.10)	0.90<br>(0.10)	0.90<br>(0.12)	0.89<br>(0.11)	0.82+<br>(0.09)
Work(to(Family Conflict	1.06<br>(0.07)	1.06<br>(0.07)	1.13+<br>(0.08)	1.11<br>(0.08)	1.11<br>(0.08)	1.07<br>(0.07)
Job insecurity	0.96<br>(0.23)	0.94<br>(0.21)	1.00<br>(0.19)	0.74<br>(0.15)	0.77<br>(0.15)	0.81<br>(0.15)
Tenure constructed	0.98+<br>(0.01)	0.99<br>(0.01)	0.99<br>(0.01)	0.98<br>(0.01)	0.98<br>(0.01)	0.99<br>(0.01)
Work Hours (this job)	0.99<br>(0.01)	0.99<br>(0.01)	0.99<br>(0.01)	0.99<br>(0.01)	0.99<br>(0.01)	0.99<br>(0.01)
Psychological Distress	1.06**<br>(0.01)	1.06**<br>(0.01)	1.05**<br>(0.01)	1.06**<br>(0.02)	1.06**<br>(0.02)	1.07**<br>(0.02)
BMI (by category)						
Normal or Underweight (BMI<25)	reference	reference	reference	reference	reference	reference
Overweight (BMI≥25 & BMI<30)	1.38+<br>(0.24)	1.35+<br>(0.22)	1.35+<br>(0.22)	1.07<br>(0.17)	1.09<br>(0.17)	1.04<br>(0.15)
Obese (BMI≥30)	1.39*<br>(0.20)	1.34*<br>(0.18)	1.38**<br>(0.17)	1.52**<br>(0.22)	1.47**<br>(0.20)	1.35*<br>(0.17)
Age	1.00<br>(0.01)	1.00<br>(0.01)	1.00<br>(0.01)	1.01<br>(0.01)	1.01<br>(0.01)	1.00<br>(0.01)
Hypertension	1.09<br>(0.16)	1.07<br>(0.15)	1.06<br>(0.13)	0.99<br>(0.15)	1.03<br>(0.15)	1.04<br>(0.14)
Smokes	1.05<br>(0.14)	1.17<br>(0.15)	1.29*<br>(0.16)	0.83<br>(0.12)	0.84<br>(0.11)	0.93<br>(0.11)
Diabetes	0.66+<br>(0.16)	0.72<br>(0.16)	0.69+<br>(0.14)	1.26<br>(0.29)	1.20<br>(0.27)	1.16<br>(0.24)

Heart Disease	0.93	0.87	0.92	0.72	0.74	0.97
	(0.45)	(0.37)	(0.34)	(0.30)	(0.28)	(0.34)
Stroke	1.71	1.48	1.39	0.59	0.81	0.71
	(0.69)	(0.58)	(0.58)	(0.29)	(0.34)	(0.30)
Hours in Bed	0.91*	0.92*	0.94+	0.93+	0.93+	0.95
	(0.04)	(0.04)	(0.04)	(0.04)	(0.04)	(0.03)
Annual Personal Income						
≤$9,999	3.05*	3.60**	2.71*	0.48	1.18	0.99
	(1.52)	(1.55)	(1.07)	(0.22)	(0.54)	(0.41)
$10,000-$19,999	1.68	1.51	1.84*	0.94	0.89	1.06
	(0.56)	(0.48)	(0.57)	(0.28)	(0.26)	(0.28)
$20,000-$29,999	1.70+	1.67+	1.79*	0.86	0.86	1.04
	(0.51)	(0.47)	(0.45)	(0.22)	(0.22)	(0.24)
$30,000-$39,999	1.60+	1.63+	1.61*	0.96	0.95	1.08
	(0.45)	(0.44)	(0.38)	(0.23)	(0.22)	(0.24)
$40,000-$49,999	1.85+	1.65	1.54	1.05	0.98	1.11
	(0.58)	(0.50)	(0.42)	(0.26)	(0.24)	(0.26)
$50,000-$59,999	1.94*	1.95*	1.78*	0.84	0.90	0.88
	(0.60)	(0.58)	(0.48)	(0.19)	(0.20)	(0.18)
>$60,000	reference	reference	reference	reference	reference	reference
Married/living with partner	0.96	0.94	0.98	0.99	0.92	0.90
	(0.13)	(0.12)	(0.12)	(0.13)	(0.12)	(0.10)
Number of children	1.07	1.04	1.01	1.04	1.05	1.00
	(0.09)	(0.07)	(0.07)	(0.08)	(0.07)	(0.06)
Household Size	0.92	0.94	0.93	0.95	0.96	1.00
	(0.06)	(0.05)	(0.05)	(0.05)	(0.05)	(0.05)
Provides care outside work	1.47**	1.39*	1.30*	0.94	0.92	1.00
	(0.20)	(0.19)	(0.16)	(0.14)	(0.13)	(0.12)
Male	0.82	0.84	0.92	0.69	0.76	0.67
	(0.21)	(0.20)	(0.19)	(0.21)	(0.21)	(0.17)
Educational Attainment						
Less than High School Graduate	0.82	0.94	0.89	0.31**	0.32**	0.24**
	(0.32)	(0.34)	(0.27)	(0.13)	(0.12)	(0.08)
High School Graduate	1.39	1.41	1.32	0.43**	0.43**	0.35**
	(0.35)	(0.34)	(0.27)	(0.10)	(0.10)	(0.07)
Some College/Technical School	1.36	1.29	1.18	0.70+	0.74	0.61**
	(0.32)	(0.29)	(0.23)	(0.14)	(0.14)	(0.10)
College Graduate	reference	reference	reference	reference	reference	reference
Constant	0.20+	0.21+	0.20*	2.11	2.00	2.25
	(0.17)	(0.17)	(0.14)	(1.62)	(1.48)	(1.43)
# Observations	2466	2659	3112	2463	2659	3112

# Individuals	822	927	1275	821	927	1275

+ p<0.10, * p<0.05, ** p<0.01

Notes: Regression also controlled for Employer insurance status, state, intervention group, and time

Table S6. Sensitivity Analyses of Number of Visits (Tomo Sample)

	(1) Sample: Original, Completed all waves, completed case analysis	(1a) Sample: Completed all waves, may have missing values	(1b) Sample: Completed at least one wave, may have missing values	(2) Sample: Original, Completed all waves, completed case analysis	(2a) Sample: Completed all waves, may have missing values	(2b) Sample: Completed at least one wave, may have missing values
	Incidence Rate Ratios (SE)	Incidence Rate Ratios (SE)	Incidence Rate Ratios (SE)	Incidence Rate Ratios (SE)	Incidence Rate Ratios (SE)	Incidence Rate Ratios (SE)
Schedule Control	0.71*	0.82	0.86	1.21	1.23+	1.15
	(0.11)	(0.13)	(0.13)	(0.14)	(0.14)	(0.13)
Above median job demands	0.72	0.79	0.80	0.83	0.82	0.84
	(0.17)	(0.17)	(0.17)	(0.12)	(0.11)	(0.11)
Below median decision authority	0.94	0.91	0.86	1.07	1.09	1.04
	(0.19)	(0.17)	(0.16)	(0.14)	(0.13)	(0.12)
Work-to-Family Conflict	1.01	0.99	1.05	1.35**	1.37**	1.40**
	(0.16)	(0.15)	(0.15)	(0.12)	(0.12)	(0.12)
Job insecurity	0.83	0.83	0.84	0.80+	0.76*	0.75*
	(0.19)	(0.17)	(0.17)	(0.10)	(0.09)	(0.09)
Tenure constructed	0.97	0.97+	0.97*	0.98*	0.98*	0.98*
	(0.02)	(0.02)	(0.02)	(0.01)	(0.01)	(0.01)
Work Hours (this job)	1.03	1.03	1.01	0.98	0.98	0.99
	(0.02)	(0.02)	(0.02)	(0.01)	(0.01)	(0.01)
Psychological Distress	0.98	1.00	1.01	1.09**	1.09**	1.07**
	(0.04)	(0.03)	(0.03)	(0.02)	(0.02)	(0.02)
BMI (by category)						
Normal or Underweight (BMI<25)	reference	reference	reference	reference	reference	reference
Overweight (BMI≥25 & BMI<30)	1.46	1.29	1.35	0.86	0.86	0.90
	(0.36)	(0.31)	(0.31)	(0.14)	(0.13)	(0.13)
Obese (BMI≥30)	1.37	1.36	1.39	1.02	1.03	1.10
	(0.37)	(0.35)	(0.34)	(0.18)	(0.17)	(0.18)
Age	1.01	1.02	1.02	1.02*	1.02*	1.02*
	(0.02)	(0.02)	(0.02)	(0.01)	(0.01)	(0.01)
Hypertension	1.20	0.99	1.01	1.23	1.28+	1.28+
	(0.30)	(0.23)	(0.22)	(0.19)	(0.18)	(0.18)
Smokes	1.87+	1.72+	1.78*	0.74	0.74	0.82
	(0.64)	(0.56)	(0.50)	(0.29)	(0.27)	(0.26)
Diabetes	1.75	1.52	1.62+	1.12	1.10	1.04
	(0.63)	(0.51)	(0.46)	(0.23)	(0.22)	(0.20)
Heart Disease	0.44	0.41	0.36	0.79	0.78	1.07
	(0.44)	(0.36)	(0.32)	(0.21)	(0.19)	(0.30)
Stroke	1.95	1.80	1.67	1.80	1.65	1.90+
	(1.71)	(1.72)	(1.55)	(0.70)	(0.65)	(0.73)
Hours in Bed	1.08	1.03	1.04	1.01	1.01	1.03

	(0.13)	(0.11)	(0.10)	(0.06)	(0.06)	(0.06)
Annual Personal Income						
≤$9,999	0.78	0.69	0.74	2.66	2.16	2.01
	(1.00)	(0.66)	(0.59)	(2.03)	(1.26)	(1.10)
$10,000-$19,999	1.37	1.36	1.36	2.42	1.79+	1.65+
	(1.45)	(0.87)	(0.87)	(1.47)	(0.56)	(0.50)
$20,000-$29,999	1.60	1.47	1.45	2.37	1.80*	1.78*
	(1.62)	(0.92)	(0.91)	(1.40)	(0.52)	(0.50)
$30,000-$39,999	0.71	0.83	0.92	2.65	2.07*	2.13**
	(0.72)	(0.53)	(0.58)	(1.57)	(0.61)	(0.61)
$40,000-$49,999	1.62	1.57	1.55	2.52	2.12*	2.45**
	(1.69)	(1.06)	(1.03)	(1.56)	(0.74)	(0.83)
$50,000-$59,999	1.45	2.04	1.51	1.10	0.81	0.79
	(2.08)	(1.97)	(1.49)	(0.93)	(0.51)	(0.41)
>$60,000	reference	reference	reference	reference	reference	reference
Married/living with partner	0.62	0.55*	0.51**	0.85	0.82	0.93
	(0.18)	(0.14)	(0.13)	(0.17)	(0.15)	(0.16)
Number of children	1.07	0.98	1.05	0.75**	0.74**	0.75**
	(0.16)	(0.13)	(0.13)	(0.08)	(0.07)	(0.07)
Household Size	1.21+	1.27**	1.26*	1.14*	1.16*	1.12+
	(0.14)	(0.12)	(0.11)	(0.07)	(0.07)	(0.07)
Provides care outside work	0.83	0.91	0.93	1.26	1.18	1.23
	(0.22)	(0.22)	(0.20)	(0.19)	(0.17)	(0.17)
Male	0.74	0.76	0.75	0.73*	0.72*	0.65**
	(0.18)	(0.17)	(0.16)	(0.11)	(0.11)	(0.09)
Educational Attainment						
Grade 12 or GED	1.67	1.03	1.00	0.78	0.74	0.87
	(0.88)	(0.56)	(0.46)	(0.41)	(0.35)	(0.32)
Some College/Technical School	0.86	0.90	0.84	1.36	1.37+	1.25
	(0.28)	(0.27)	(0.25)	(0.27)	(0.26)	(0.23)
College Graduate	reference	reference	reference	reference	reference	reference
Constant	0.05	0.04+	0.04+	0.04**	0.05**	0.05**
	(0.11)	(0.08)	(0.07)	(0.05)	(0.05)	(0.05)
# Individuals	490	1640	1806	490	1639	1805
# Observations	1470	582	695	1470	582	695

+ p<0.10, * p<0.05, ** p<0.01

Notes: Job category, Intervention group, time, randomization, and a merger indicator were also included in the model.
